# Déclenchement du travail à terme par le misoprostol: expérience d'une maternité tunisienne

**DOI:** 10.11604/pamj.2016.24.28.8141

**Published:** 2016-05-09

**Authors:** Nadia Ouerdiane, Nihel Tlili, Kaouther Othmani, Walid Daaloul, Abdelwaheb Masmoudi, Sonia Ben Hamouda, Badreddine Bouguerra

**Affiliations:** 1 Service de Gynécologie Obstétrique B, Hôpital Charles Nicolle Tunis, Faculté de Médecine de Tunis, Université Tunis El Manar, Tunis, Tunisie

**Keywords:** Misoprostol, déclenchement du travail, efficacité, morbidité, Misoprostol, induction of labour at term, efficiency, morbidity

## Abstract

Evaluer l'efficacité et l'innocuité de l'utilisation du misoprostol par voie vaginale pour le déclenchement du travail à terme. Etude prospective réalisée au service de gynécologie obstétrique B de l'hôpital Charles Nicolle de Tunis sur une durée de 4 mois. La population sélectionnée concernait les patientes à terme devant bénéficier d'une maturation cervicale. Le misoprostol à la dose de 50 µg par voie vaginale toutes les 12 h était utilisé. Les paramètres étudiés étaient les anomalies contractiles, les anomalies du RCF, le mode d'accouchement, le délai d'accouchement et l’état néonatal. 44 patientes ont bénéficié d'une maturation cervicale par misoprostol. Le terme moyen était de 40 SA. Le taux de nullipare était de 23/44 (52%). Le taux d'accouchement par voie basse était de 31/44 (70.4%). 84% des patientes ont reçu une seule dose de misoprostol. Les anomalies du RCF ont été notées dans 14/44 (32%). Le taux de liquides méconiaux était de 12/44 (27%). Un score d'Apgar à 5 mn inférieur à 7 était noté chez 7/44 (16%). Un cas de rupture utérine était survenue chez une primipare et ce après une seule prise de misoprostol. Nos résultats sont décevants en raison de la survenue d'une rupture utérine et d'une morbidité néonatale importante. D'autres études prospectives multicentriques restent utiles pour mieux s'assurer de l'efficacité mais surtout de l'innocuité du misoprostol à dose faible pour le déclenchement du travail à terme.

## Abstract (en)

To evaluate the efficacy and safety of vaginal misoprostol for term labour induction. A prospective study conducted at the Department of Obstetrics and Gynecology B of hospital Charles Nicolle, Tunis, over a period of 4 months. The group of subjects, selected to represent the population of interest, were pregnant patients at term undergoing cervical ripening. Patients received 50 mcg vaginal misoprostol every 12 hours. The parameters studied were: contractile abnormalities, abnormalities of fetal heart rate (FHR), mode of delivery, delayed delivery and neonatal status. 44 patients underwent cervical ripening with misoprostol. The average term was 40 WA. Nulliparous rate was 23/44 (52%). Vaginal birth rate was 31/44 (70.4%). 84% of patients received a single dose of misoprostol. FHR abnormalities were observed in 14/44 (32%). The rates of meconium-stained amniotic fluid was 12/44 (27%). Apgar score of less than 7 at 5 minutes was found in 7/44 (16%). A case of uterine rupture occurred in a primipara after a single dose of misoprostol. Our results are disappointing due to the occurrence of 1 uterine rupture and of 1 significant neonatal morbidity. Other multicentre prospective studies will be useful to better ensure the effectiveness but primarily the safety of low-dose misoprostol for induction of labour at term.

## Introduction

Le déclenchement artificiel du travail est l'une des procédures les plus fréquentes chez la femme enceinte. Le plus souvent, l'indication est posée sur un col immature. L'utilisation des prostaglandines est indispensable en cas de maturation sur col défavorable. La dinoprostone est la molécule la plus prescrite dans cette indication [1]. Le misoprostol est aujourd'hui un concurrent de la dinoprostone malgré l'absence de l'AMM dans cette indication. Il s'agit d'un analogue synthétique des prostaglandines E1 [2]. Il est donc indispensable de mesurer précisément le rapport bénéfices/risques de la prescription de cette molécule en privilégiant le dosage associé à la plus grande innocuité.

## Méthodes

Il s'agit d'une étude prospective réalisée dans la maternité du niveau III de l'hôpital Charles Nicolle de Tunis, sur une période de 4 mois allant 1^er^ octobre 2013 au 26 janvier 2014. Les données ont été recueillies de façon prospective à partir des dossiers des patientes. Les critères d'inclusion comprenaient les situations présentant une indication médicale de déclenchement, il s'agissait de grossesses monofoetales, à terme, en présentation céphalique avec des conditions cervicales défavorables (score de Bishop < 6). Pour toutes les patientes, l'indication du déclenchement et la décision d'administration du misoprostol étaient discutées en équipe. Concernant le déroulement du protocole, le misoprostol était mis dans le cul de sac vaginal après un enregistrement cardiaque fœtal. Le score de Bishop était noté lors de la première pose et à chaque évaluation du col. Notre protocole était d'administrer par voie vaginale du misoprostol à la dose de 50 µg toutes les 12 heures sans dépasser 3 prises. Les patientes non entrées en travail après les 3 doses étaient réévaluées: si le score de Bishop était favorable, un déclenchement par ocytocine était réalisé après l'amniotomie. En cas d’échec de maturation, une césarienne était réalisée. Dans cette étude, les paramètres étudiés étaient les anomalies de contractions utérines, le taux de liquide méconial, le taux de césariennes, le taux de score d'Apgar < 7 à 5 minutes, le taux d'admission en réanimation néonatale et le taux de rupture utérine.

## Résultats

La population étudiée était constituée de 44 cas de déclenchement par Cytotec^®^. Les caractéristiques maternelles sont présentées dans le Tableau 1. Le taux de nullipare était de 23/44 (52%). Le score de Bishop moyen avant la maturation était 1.56. L'indication du déclenchement était la grossesse prolongée dans 21/44 (48%), le diabète gestationnel dans 10/44 (22.7%), l'oligoamnios dans 8/44 (18%) et l’ HTA dans 5/44 (11.4%). Quatre-vingt-quatre pourcent des patientes ont reçu une seule dose de misoprostol (50 µg). Le recours à 3 doses était noté dans 3/44 (6.8%). Le taux d'accouchement par voie basse dans les 24 heures était de 31/44 (70.4%). L'indication de césarienne était dominée par la souffrance fœtale aigue dans 10/44 (22.7%). Le délai moyen d'accouchement après la première prise de 17 h avec des extrêmes variant de 4 à 40 heures. Le recours aux ocytociques était observé dans 13/44 (29.5%). Les anomalies du RCF observées dans 14/44 (32%) étaient représentées essentiellement par les décélérations. Les anomalies contractiles à type d'hypercinésie ont été observées dans 7/44 (16%). Concernant les résultats néonatals (Tableau 2), le taux de liquides méconiaux était évalué à 12/44 (27%), quatre des 44 enfants (16%), avaient un score d'Apgar inférieur à 7 à 5 minutes. Nous avons noté 10/44 (22.7%) admissions en soins intensifs pédiatriques avec bonne évolution dans tous les cas. Concernant la morbidité maternelle, les résultats sont présentés dans le Tableau 3. Il y a eu 3 cas d'inertie utérine (6.8%) survenues au cours des accouchements par voie basse et ayant nécessité la perfusion du Nalador^®^. Un cas de rupture utérine sur utérus sain est survenu chez une primipare âgée de 28 ans hospitalisée pour grossesse prolongée. Le diagnostic de rupture était retenu en per opératoire lors de la césarienne réalisée pour SFA 8 heures après la première prise de cytotec^®^. Le nouveau-né a pesé 3350 g et avait un bon Apgar. Nous avons réalisé un traitement conservateur et les suites opératoires étaient simples.

**Tableau 1:** Caractéristiques des patientes bénéficiant d'une maturation cervicale par misoprostol

Variables (moyenne et extrêmes)	n= 44
Age maternel	30 (20-41)
Gestité	1 (1-5)
Parité	1.7 (1-3)
Nulliparité n (%)	23 (52)
Age gestationnel en SA	40 (37-42)
Score de Bishop initial	
Bishop 0-2 n (%)	36 (82)
Bishop 3-4 n (%)	8 (18)
Nombre de prises de Cytotec^®^	
1 dose n (%)	37 (84%)
2 doses n (%)	5 (11.3)
3 doses n (%)	2 (4.7)

**Tableau 2:** Résultats néonataux

Variables (moyenne et extrêmes)	n= 44
Poids de naissance	3300 (2000-4650)
Apgar	
5 minutes	8.2 (2-9)
10 minutes	9.9 (6-10)
Apgar < 7 n (%)	
5 minutes	7 (16)
10 minutes	3 (6.8)
Liquide méconial n (%)	12 (27)
Séjour en réanimation n (%)	10 (22.7)

**Tableau 3:** Résultats concernant la morbidité maternelle

Variables (nombre,%)	n= 44
Hypercinésie	7 (16)
Inertie utérine	3 (6.8)
Recours aux Nalador^®^	3 (6.8)
Transfusion sanguine	0
Rupture utérine	1 cas

## Discussion

Le Cytotec^®^ (misoprostol) est un analogue de PGE1 utilisé dans le traitement de l'ulcère gastroduodénal. Les avantages de cette molécule sont le prix modique (inférieur à un euro le Comprimé de 200 µg), la facilité de conservation et d'utilisation [3]. Malgré l'absence d'AMM, ce produit est largement utilisé dans les interruptions volontaires de grossesse, les interruptions médicales de grossesse et en cas de mort fœtale in utero. La posologie est alors variable selon les centres, par voie orale ou vaginale [4]. Selon la méta-analyse de Hofmeyer [5], le misoprostol est une molécule plus efficace que la dinoprostone intra vaginale. Sur 13 essais randomisés (2906 patientes), il a été démontré que le taux d'accouchement par voie vaginale de plus de 24 heures est diminué de 20% avec le misoprostol. De même, la nécessité de recours à l'ocytocine était nettement réduite. Toutefois, le taux de césarienne n'a pas diminué de façon significative. Dans notre étude, le taux d'accouchements par voie basse était de 31/44 (70.4%) et toutes ces patientes ont accouché dans les premières 24 heures suivant la première prise de Cytotec^®^. D'autre part, le misoprostol pourrait être considéré comme une molécule plus dangereuse que la Dinoprostone vaginale [6]. En effet les paramètres qui ont été étudiés étaient le nombre de décès périnataux, le taux de rupture utérine, le taux d'hospitalisation en réanimation néonatale ainsi que le taux d'Apgar < 7 à cinq minutes. Ces résultats concernent surtout des travaux réalisés avec des doses élevés de misoprostol (100 µg soit demi comprimé) et sont dus à l'augmentation significative des anomalies contractiles [7]. En effet, le misoprostol exerce une plus forte activation sur les cellules myométriales. Pour l'utilisation sécurisée du misoprostol comme agent maturant à terme, il faut bien choisir la population. Il faut à l’évidence contre-indiquer l'utilisation de misoprostol en cas d'utérus cicatriciel en raison de l'effet contracturant élevé de la molécule [8]. Concernant la voie d'administration, nombreuses études ont montré une différence significative entre la voie vaginale et la voie orale et ce concernant la cinétique plasmatique du misoprostol. Le maximum du pic plasmatique est obtenu 34 minutes après prise orale versus 80 mn après prise vaginale. Toutefois, la concentration sérique reste élevée plus longtemps par voie vaginale. Pour cela, la voie vaginale est actuellement préférée et privilégiée [9–11]. Pour le choix de la dose du misoprostol et l'intervalle de pose et compte tenu du risque important d'hyperstimulation utérine, les différentes études ont conclu à la nécessité de réduire les doses de misoprostol et d'augmenter l'intervalle de pose [12, 13]. D'après le travail de Wing [14] qui a fait varier la dose et l'intervalle de pose de misoprostol, il a été démontré que la dose de 25 µg toutes les 6 heures a moins d'effets secondaires à type d'hypercinésie et une efficacité similaire à la dinoprostone.

Dans notre étude l'intervalle de pose était suffisamment long (12 heures) mais la dose était élevée (50µg). Notre travail a été arrêté lors de la survenue d'une rupture utérine. Plusieurs études [13, 14] ont comparé l'utilisation du misoprostol à la dose de 25 et de 50 µg administrées toutes les 4 heures. Elles retrouvent que le taux d'accouchement dans les 24 heures était proche mais le nombre de césariennes pour anomalies du RCF était significativement supérieur dans le groupe 50 µg. Sur notre échantillon, les anomalies du RCF ont été observées dans 14/44 (32%). En comparaison avec le Dinoprostone, Ramsey [15] a récemment montré lors d'une étude rétrospective randomisée que le misoprostol à la dose de 50 µg toutes les 6 heures donnerait plus d'anomalies contractiles et d'anomalies du RCF que la dinoprostone sous forme de gel intracervical (Prépédil^®^) administrée toutes les 6 heures. Concernant l'efficacité du misoprostol à la dose de 25 µg/ 4h versus celle du dinoprostone, 3 essais randomisés ont été publiés [16–18]. Le délai du déclenchement était comparable dans les 2 groupes, une tendance de moins de césariennes dans le groupe misoprostol était retrouvée. La différence n’était pas significative entre les 2 groupes concernant les anomalies du RCF et celles des contractions utérines. Par ailleurs, le taux de score d'Apgar <7 à 5mn et le taux d'hospitalisation en réanimation néonatale était nettement moindre dans le groupe misoprostol 25 µg/4h (19%) vs 26% dans le groupe dinoprostone. La dose de misoprostol de 25 µg/ 4h par voie vaginale semble être la posologie de référence [13, 14, 16, 17]. Avec cette posologie et ce rythme d'administration, on semble avoir le meilleur compromis innocuité-efficacité pour la voie vaginale. En pratique clinique, nous ne disposons que de comprimés de 200 µg, si le 1/4 de comprimé pourrait être obtenu, le 1/8 de comprimé soit 25 µg est impossible à obtenir. Il se pose donc le problème de forme galénique du misoprostol à utiliser par voie vaginale. A Strasbourg, les médecins utilisent depuis octobre 2000 le Cytotec^®^ sous forme de gélules dosées à 25 µg comportant le produit sous forme de gel [8–13]. En plus, il existe d'autres avantages en faveur de l'utilisation du misoprostol, il s'agit d'un produit dont la conservation peut se faire à température ambiante. Le deuxième avantage est celui du coût qui reste nettement réduit par rapport au coût de la dinoprostone [19].

## Conclusion

Bien que le misoprostol par voie vaginale est supérieur à la dinoprostone en terme d'efficacité et du coût, mais en terme d'innocuité, on se doit de rester prudent en cas d'utilisation hors AMM de ce produit. Les essais randomisés publiés récemment démontrent un taux moindre d'anomalies contractiles et du RCF avec des doses faibles de misoprostol (25µg) par rapport à la dose de 50 µg et par rapport à la prostaglandine de référence la dinoprostone.

### Etat des connaissances actuelle sur le sujet

- Le misoprostol est une molécule plus efficace que la dinoprostone intra vaginale pour le déclenchement du travail à terme.

### Contribution de notre étude à la connaissance

- En terme d'innocuité et en raison d'une morbidité néonatale importante décrite dans notre série, on se doit de rester prudent et de s'assurer à travers d'autres études prospectives de l'innocuité du misoprostol pour le déclenchement du travail à terme.

## Conflits d'intérêts

Les auteurs ne déclarent aucun conflit d'intérêts.

## Contributions des auteurs

Tous les auteurs cités dans la page de titre ont contribué à la conception, l'acquisition de données, ou l'analyse et l'interprétation des données ainsi qu’à la rédaction de l'article. Tous les auteurs ont contribué à la conduite de ce travail. Tous les auteurs déclarent également avoir lu et approuvé la version finale du manuscrit.
